# Efficacy of antifibrotic treatment for ANCA-positive fibrosing interstitial lung disease: a retrospective case‒control study

**DOI:** 10.1186/s12890-026-04109-1

**Published:** 2026-01-22

**Authors:** Zhiyi Li, Ruxuan Chen, Qingyang Liu, Hui Huang, Chi Shao, Zuojun Xu

**Affiliations:** 1 Department of Internal Medicine, Peking Union Medical College Hospital, Chinese Academy of Medical Sciences & Peking Union Medical College, #1 Shuaifuyuan Street, Dongcheng District, Beijing, China; 2 Department of Pulmonary and Critical Care Medicine, Peking Union Medical College Hospital, Chinese Academy of Medical Sciences & Peking Union Medical College, #1 Shuaifuyuan Street, Dongcheng District, Beijing, China; 3 Pharmacy Department, Peking Union Medical College Hospital, Chinese Academy of Medical Sciences & Peking Union Medical College, #1 Shuaifuyuan Street, Dongcheng District, Beijing, China

**Keywords:** ANCA, Fibrosing interstitial lung disease, Antifibrotic treatment, FVC

## Abstract

**Introduction:**

Studies verifying the performance of antifibrotic drugs for the treatment of ANCA positive fibrotic ILD (ANCA-fILD) are lacking.

**Materials and methods:**

This study assessed the clinical features of ANCA-fILD patients with or without add-on treatment with antifibrotic drugs. A retrospective study involving ANCA-positive patients treated from January 2012 to December 2023 at Peking Union Medical College Hospital was conducted, and a case‒control analysis was performed accordingly.

**Results:**

A total of 105 ANCA-fILD patients were identified from 18,617 ANCA-positive patients treated from January 2012 to December 2023 at Peking Union Medical College Hospital; these patients were further divided into an add-on group (31 patients) and a non-add-on group (74 patients). On the basis of baseline FVC%pred matching (± 10%), 30 and 60 patients were ultimately included in the add-on and non-add-on groups, respectively. The decreases in pFVC (*p* = 0.03), FVC (*p* = 0.02), DLCO (*p* = 0.01), and pDLCO (*p* < 0.01) were less significant in the add-on group than in the non-add-on group. In the related sample analysis, the add-on group did not present significant differences in FVC (*p* = 0.47), pFVC (*p* = 0.53), DLCO (*p* = 0.90), and pDLCO (*p* = 0.43) between baseline and at the end of the 1-year follow-up, whereas the non-add-on group presented significant decreases in FVC (*p* < 0.01), pFVC (*p* < 0.01), DLCO (*p* < 0.0001) and pDLCO (*p* < 0.0001). The incidence rates of adverse events and acute exacerbations were similar between the two groups.

**Conclusion:**

Add-on antifibrotic medications seem to effectively slow the deterioration of lung function in patients with ANCA^+^ fILD without causing any obvious new-onset adverse reactions.

**Supplementary Information:**

The online version contains supplementary material available at 10.1186/s12890-026-04109-1.

## Highlights

1) Preserved lung function: add-on antifibrotic therapy significantly attenuated the decline in vital capacity and diffuse function over 1 year compared to the non-add-on group.

2) Safety Profile: No significant differences in adverse events or acute exacerbations were observed between groups, supporting the safety of antifibrotics in ANCA-fILD.

3) Clinical Relevance: This is the first large-scale retrospective analysis (*n* = 105) to specifically examine antifibrotic use in ANCA-fILD, with case-control matching for baseline FVC%pred.

**Supplementary Information:**

The online version contains supplementary material available at 10.1186/s12890-026-04109-1.

## Introduction

Interstitial lung disease (ILD) is a group of diseases characterized by diffuse lung parenchymal injuries, for which different treatment strategies should be applied according to different disease behaviors [1]. Fibrosing interstitial lung disease (fILD) is characterized by pulmonary fibrosis, inflammation, and proliferation of the pulmonary alveolar wall [2–5]. Among the various types of fILD, idiopathic pulmonary fibrosis (IPF) is the most common form of fILD. High-resolution computed tomography (HRCT) of the chest commonly reveals a usual interstitial pneumonia (UIP) pattern, characterized by reticular opacities and honeycombing predominant in the lower lobe [6]. Moreover, the common causes of fILD [2] also include systemic autoimmune rheumatic disease-ILD (SARD-ILD) and chronic hypertensive pneumonitis. Except for UIP, fibrotic nonspecific interstitial pneumonia pattern (fNSIP) is also observed in cases of fILD and is characterized by bilateral ground-glass opacities mainly in the mid-to-lower lung zone, with fine reticulation or traction bronchiectasis [1].

During follow-up examinations, patients with ILD can test positive for anti-neutrophil cytoplasmic antibodies (ANCAs) [7–9], targeted to proteins within the cytoplasm of neutrophils. There are different methods to test for ANCAs [10], including a chemiluminescence immunoassay (CLIA) and the immunofluorescence technique (IF). Depending on the different types of targeting antigens used in a CLIA, the results can reveal myeloperoxidase-ANCA (MPO-ANCA) and proteinase 3-ANCA (PR3-ANCA). The IF technique can reveal perinuclear ANCA (p-ANCA) and cytoplasmic ANCA (c-ANCA). Traditionally, ANCAs are thought to be related to systematic vasculitis, known as ANCA-associated vasculitis (AAV). However, clinical observations indicate that patients with patterns indicating fILD and ANCA positivity (ANCA-fILD) are unlikely to progress to AAV [11, 12]. In addition, these patients have a poor response to anti-inflammatory and immunosuppressive treatments and even experience progression due to immunosuppression treatment [9, 13]. In contrast, some studies have even proven the efficacy of antifibrotic treatment for ANCA-positive fILD (ANCA-fILD) on the basis of the treatment delaying pulmonary function decline and improving patient prognosis. Although clinical experience and several real-world studies have confirmed the efficacy and safety of antifibrotic drugs for systemic autoimmune rheumatic disease (SARD)-ILD and SARD-progressive pulmonary fibrosis (PPF) [14, 15], studies verifying the performance of antifibrotic drugs for the treatment of ANCA-fILD are lacking. Nevertheless, the significance of fILD among ANCA-ILD patients is nonnegligible. Therefore, in our retrospective study and case‒control analysis, we evaluated the effect of antifibrotic treatment in ANCA-fILD patients, aiming to provide more solid evidence for treatment strategies for ANCA-fILD patients.

## Materials and methods

### Study design

This study was approved by the Ethics Committee of Peking Union Medical College Hospital (I-22PJ1130). This study was designed as a matched case‒control study to determine the potential importance of antifibrotic treatment for fILD in patients positive for ANCAs. For each patient, 2 controls were matched on the basis of pFVC (± 10%) using the R program.

### Study participants

From January 2012 to December 2023, 18,617 patients tested positive for ANCAs at Peking Union Medical College Hospital (PUMCH), among which 786 patients presented with pulmonary parenchymal association. According to consensus in *European respiratory journal*, fILD is characterized by fibrosis detected on pathology, or by the presence of honeycombing and/or traction bronchiectasis on chest high-resolution computed tomography (HRCT). In our study, the diagnosis of fILD was conducted by experienced pulmonologists according to the chest HRCT. The following information was recorded during the follow-up: (1) demographic, serologic and information; (2) treatment regimen; (3) chest HRCT pattern; (4) pulmonary function test (PFT) result; (5) drug-related side effects; and (6) the number of acute exacerbation events during the 12-month follow-up period.

### Sample collection and measurements

Baseline information was collected at the beginning of the follow-up, and the following information was analyzed: demographic information (age, sex), serologic information (type of ANCAs), chest CT pattern, and pulmonary function test results. For the add-on group, the baseline was defined as the date when antifibrotic treatment was initiated, and for the non-add-on group, the baseline was defined as the date of pulmonary function test used for matching with the add-on group. For the serologic test, immunofluorescence was used to test for p-ANCAs and c-ANCAs, whereas a chemiluminescence immunoassay was used to test for MPO-ANCAs and PR3-ANCAs. Furthermore, we recorded acute exacerbation events in patients with ILD and side effects associated with the drugs during the 1-year follow-up. In addition, at the end of the 1-year follow-up, we recorded whether the patients satisfy the diagnosis of ANCA-associated vasculitis.

All patients underwent serial chest HRCT examinations in our hospital at the beginning and end of the follow-up. All CT images were independently reviewed by two respiratory physicians (each with ≥ 10 years of ILD-specific practice), with adjudication for discordant cases. On chest HRCT, the characteristic features of fILD include the presence of fibrosis, including honeycombing and/or traction bronchiectasis. The patterns on HRCT were classified into UIP and fNSIP according to differences in the performance of HRCT. According to the 2018 ATS/ERS/JRS/ALAT guideline [16], the UIP is categorized as UIP, probable UIP or indeterminate UIP. In the event that a fNSIP is observed, in addition to characteristic ground-glass opacities, there should be predominant fibrotic components, such as reticular opacities, thickening of bronchovascular bundles and traction bronchiectasis.

All patients underwent pulmonary function tests (PFTs) at the beginning and end of the 12-month follow-up. The following measurements were retrieved: (1) forced expiratory volume in 1 s (FEV_1_) and FEV_1_ as a percentage of the predicted value (pFEV_1_); (2) forced vital capacity (FVC) and FVC as a percentage of the predicted value (pFVC); (3) FEV_1_/FVC; (4) the single-breath diffusing capacity of the lung for CO adjusted by hemoglobin level (DLCO) and DLCO as a percentage of the predicted value (pDLCO); and (5) total lung capacity (TLC) and TLC as a percentage of the predicted value (pTLC).

### Statistical analysis

The primary outcome was the change from baseline pFVC in each group, presented as the △pFVC, which was equal to the pFVC (at the end of the follow-up) minus the absolute pFVC (at baseline). In addition, we also evaluated other key secondary outcomes in the study: (1) △FVC: the annual decline in absolute FVC; (2) △TLC: the annual decline in absolute TLC; and (3) △pTLC: the annual decline in pTLC. (4) △DLCO: the annual decline in absolute DLCO; (5) △pDLCO: the annual decline in pDLCO; (6) the number of acute exacerbation events during the follow-up; (7) paired comparison of FVC in each group; (8) paired comparison of pFVC in each group; (9) paired comparison of TLC in each group; (10) paired comparison of pTLC in each group; 11) paired comparison of DLCO in each group; 12) paired comparison of pDLCO in each group; and 13) the number and category of side effects during the follow-up.

Continuous variables were assessed for normality using Shapiro‒Wilk tests (α = 0.05) and visual inspection of Q-Q plots. Normally distributed data are presented as means ± standard deviations (SDs), whereas nonnormally distributed variables are reported as medians (interquartile ranges [IQRs]). Categorical variables are expressed as counts (percentages). Between-group comparisons were performed using independent samples t tests (for normally distributed variables with homogeneity of variance confirmed by Levene’s test, Mann‒Whitney U tests (for nonparametric continuous data), and Fisher’s exact tests (for categorical variables). Paired measurements within each group were performed by paired t tests (for normally distributed data) and Wilcoxon signed-rank tests (for nonparametric data). To address potential immortal time bias, we performed a secondary time-dependent Cox proportional hazards analysis in which antifibrotic treatment was treated as a time-varying covariate. Patients were considered untreated until the date of treatment initiation, after which they were considered treated in the analysis. A p value < 0.05 indicated statistical significance. All statistical data were analyzed with Prism 10.

## Results

### Patients’ characteristics

**Fig. 1:**
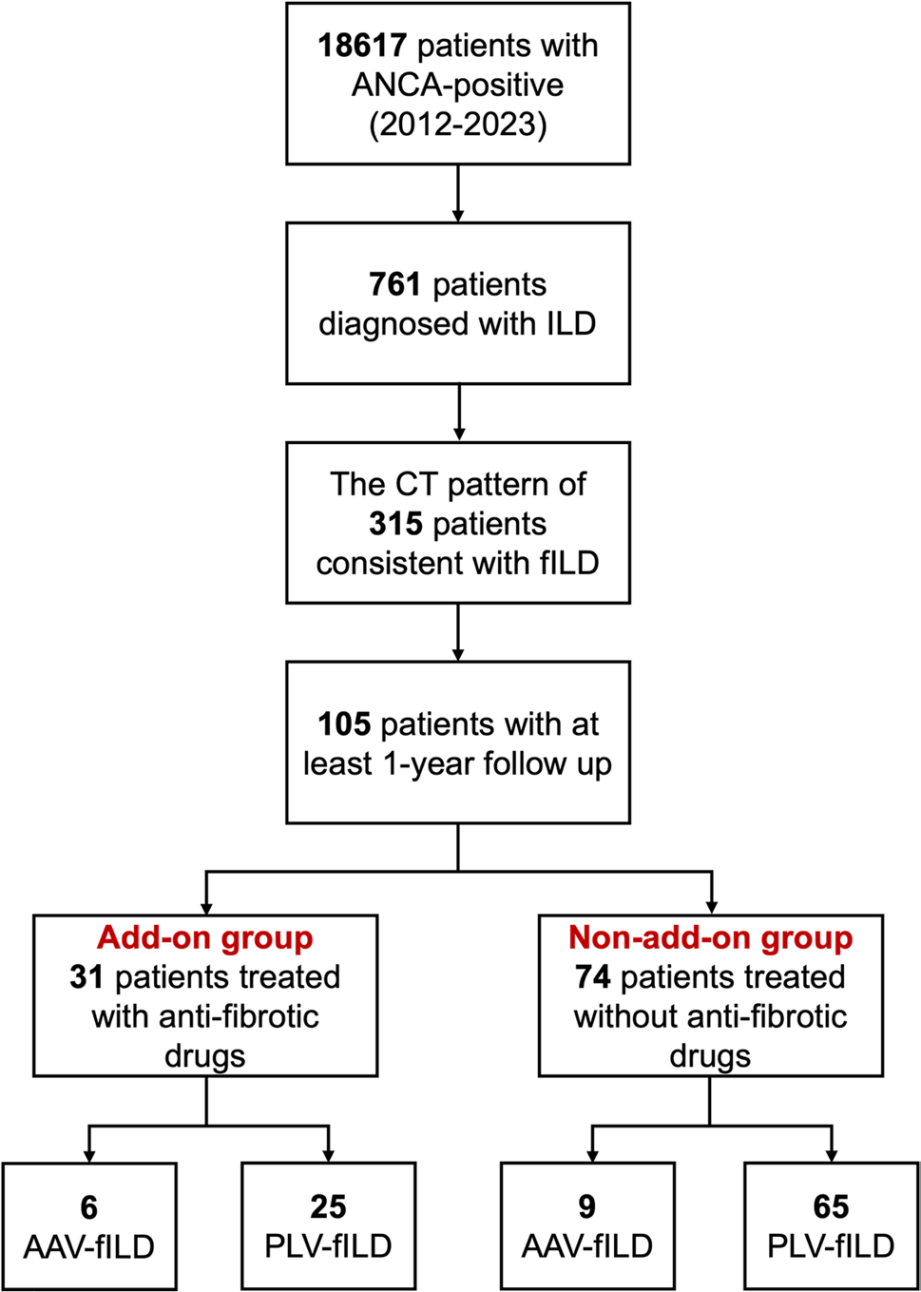
The flowchart of patients in this study

After thorough evaluation of their chest CT patterns by two experienced pulmonologists, a total of 105 patients with ANCA-positive fibrotic interstitial lung disease (ANCA-fILD) were included in this study. Among them, 31 patients were treated with add-on antifibrotic drugs during the entire follow-up period (add-on group), while the remaining 74 patients did not receive any antifibrotic treatment during the follow-up period (non-add-on group). Our cohort included patients with isolated ANCA-positive fILD but no clinical evidence of systemic vasculitis (termed as pulmonary limited vasculitis with fibrotic ILD, PLV-fILD), and patients with established diagnosis of AAV with predominant fibrotic ILD pattern on HRCT (termed as AAV-fILD). In the add-on group, there were 6 patients diagnosed as AAV-fILD and 25 patients as PLV-fILD. Of the 31 patients, 6 were treated with nintedanib, and 25 were treated with pirfenidone (Fig. 1). In the non-add-on group, there were 9 patients were diagnosed as AAV-fILD and 65 patients as PLV-fILD.

**Fig. 2:**
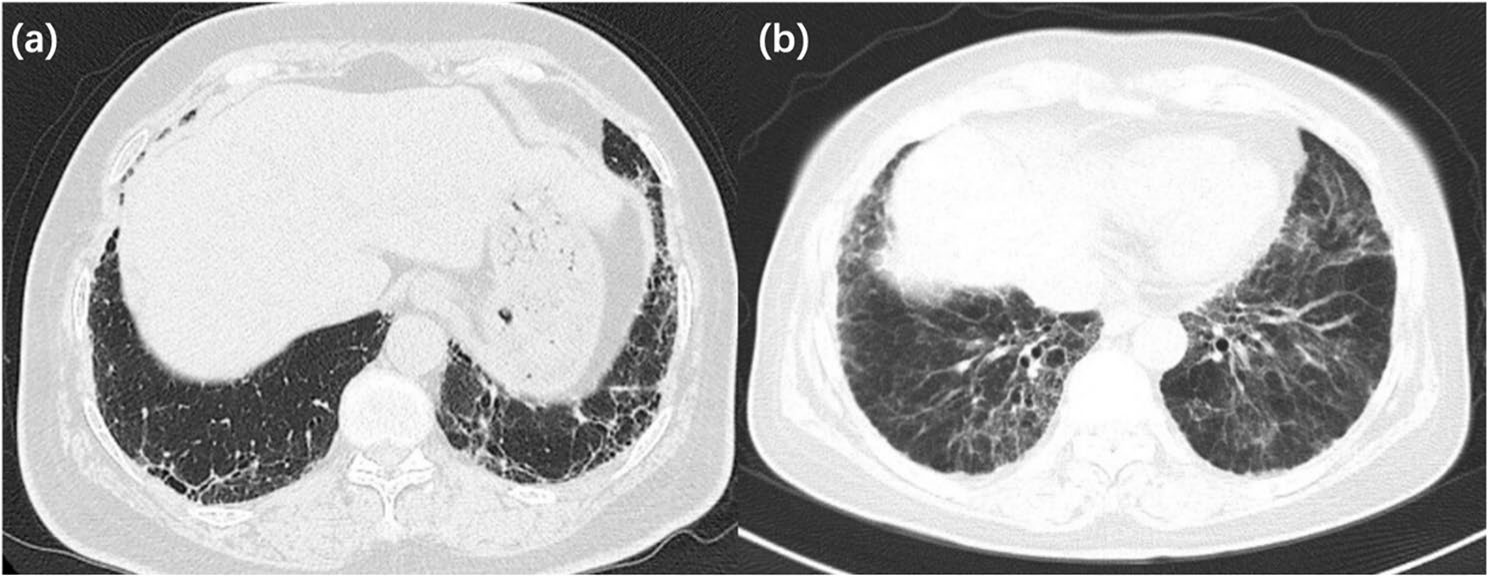
The typical UIP pattern (**a**) and NSIP patten (**b**) on chest CT pattern in this study

Thirty groups of patients were ultimately included in the study (Table 1). The average age in the add-on group [66.00 (11.25)] was similar (*p* = 0.86) to that in the non-add-on group [64.50 (11.75)]. In addition, the two groups presented similar sex distributions (*p* = 0.50) and serological features for p-ANCA (*p* = 0.15), c-ANCA (*p* = 0.47), MPO-ANCA (*p* = 0.25) and PR3-ANCA (*p* = 1.00), while p-ANCA positivity was predominant in both groups. The pFVC was similar at baseline, providing comparability for subsequent statistical evaluation. Four patients in the non-add-on group did not complete the 1-year pulmonary function test; therefore, the total number of patients included in PFT analysis for non-add-on group was 56. Most patients in both groups presented with a UIP pattern (add-on vs. non-add-on, 86.67% vs. 81.67%, *p* = 0.77) on chest HRCT (Fig. 2), with a reticular pattern in the periphery of the lower lungs and typical honeycomb patterns. As for smoking history, there was no significant difference between the two groups (30.00% vs. 35.00%, *p* = 0.82). Regarding concomitant medication, glucocorticoids (*p* = 0.04) and immunosuppressants (*p* < 0.01) were predominantly applied in patients without antifibrotic treatment. Interestingly, only a minority (add-on vs. non-add-on, 16.67% vs. 8.33%, *p* = 0.17) of patients had progressed to ANCA-associated vasculitis at the final follow-up. In terms of the type of antifibrotic drugs used, 25 patients (83.33%) used pirfenidone, and 5 patients (16.67%) used nintedanib.

**Table 1:** Demographic information

Project	Add-on group *N* = 30	Non-add-on group *N* = 60	Statistic value	*P* value
Age	66.00(11.25)	64.50(11.75)	U = 879	0.86
Gender				
Male	18(60.00%)	30(50.00%)		0.50
Female	12(40.00%)	30(50.00%)		
Type of ANCA antibody				
p-ANCA	22(73.33%)	52(86.67%)		0.15
c-ANCA	4(13.33%)	5(8.33%)		0.47
MPO-ANCA	8(26.67%)	24(40.00%)		0.25
PR3-ANCA	1(3.33%)	4(6.67%)		1.00
PFT at base line				
pFEV_1_(%)	83.93 ± 18.50	84.11 ± 15.78	T = 0.05	0.96
pFVC(%)	83.30 ± 19.26	84.90 ± 18.05	T = 0.39	0.70
pDLCO(%)	48.61 ± 14.10	59.44 ± 17.20	T = 2.98	< 0.01
pTLC(%)	72.40 ± 15.77	74.16 ± 13.41	T = 0.55	0.58
Chest HRCT pattern				
UIP	26(86.67%)	49(81.67%)		0.77
fNSIP	4(13.33%)	11(18.33%)		0.77
AAV	5(16.67%)	5(8.33%)		0.29
Smoking history	9(30.0%)	21(35.00%)		0.82
Concomitant medication				
glucocorticoids	18(60.00%)	48(80.00%)		0.04
immunosuppressants	17(56.67%)	52(86.67%)		< 0.01
Presence of PPF	1(3.33%)	1(1.67%)		1.00

*ANCA* Anti-neutrophil cytoplasmic antibodies, *MPO* Myeloperoxidase, *PR3* Proteinase 3, *PFT* Pulmonary function test, *pFEV1* forced expiratory volume in 1 s as a percentage of the predicted value, *pFVC* forced vital capacity as a percentage of the predicted value, *pDLCO* the single-breath diffusing capacity of the lung for CO adjusted by hemoglobin level as a percentage of the predicted value, *pTLC* total lung capacity as a percentage of the predicted value, *HRCT* High-resolution computer tomography, *UIP* Usual interstitial pneumonia, *fNSIP* fibrotic non-specific interstitial pneumonia, *AAV* Anca-associated vasculitis

**Fig. 3:**
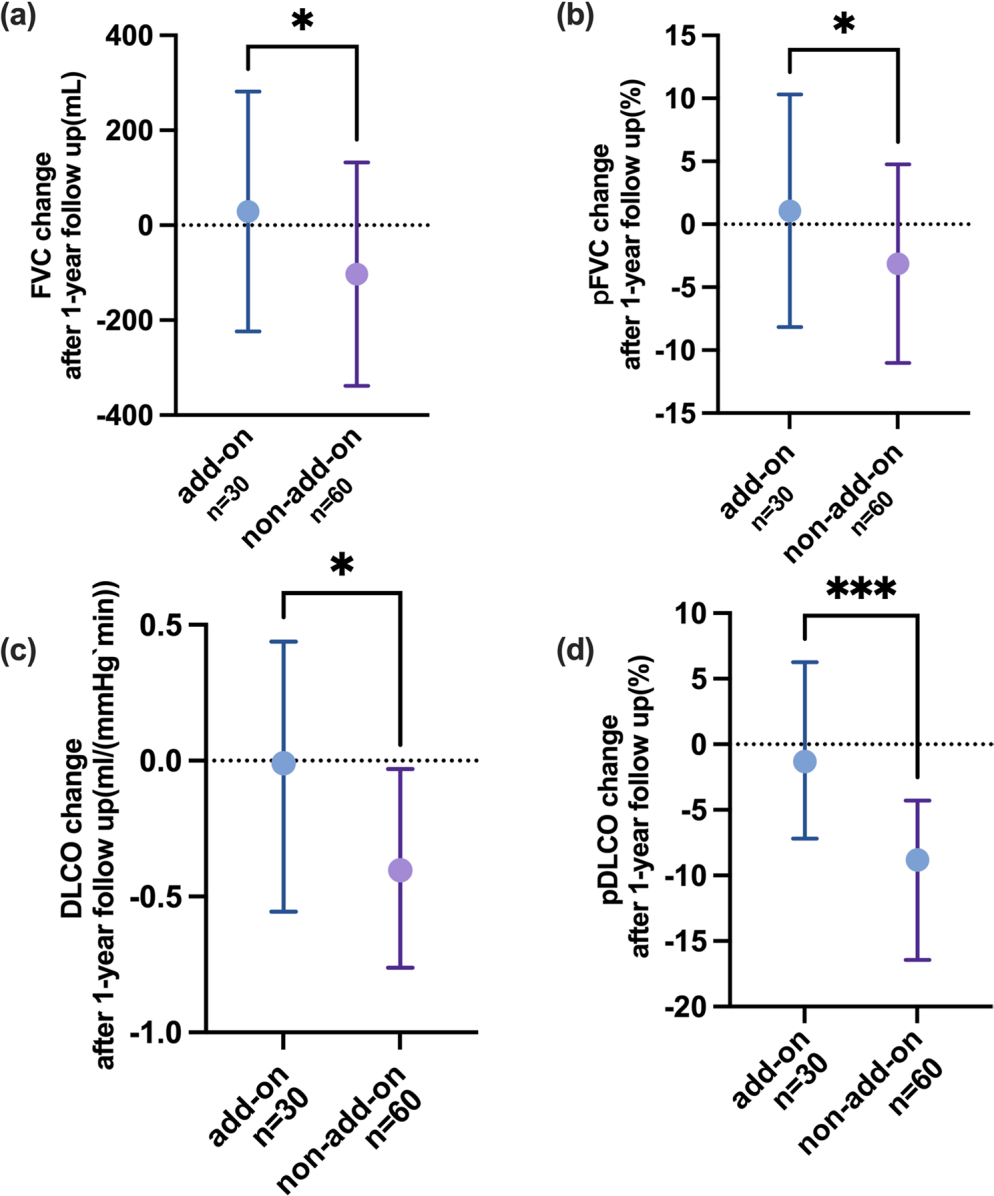
The comparison of △pFVC (**a**), △FVC (**b**) and △TLC (**c**) through the follow-up between the groups. Normally distributed data (FVC and pFVC change, Fig. 1(a) and 1(b)) are presented as means ± SD, whereas nonnormally distributed variables (DLCO and pDLCO change, Fig. 1(c) and 1(d)) are reported as medians (IQR). *: *p* < 0.05, **: *p* < 0.01, ***: *p* < 0.001

### PFT measurements

We compared the decreases in pFVC in both groups at the 1-year follow-up (Table 2 and Fig. 3). The results revealed that the change in pFVC in the non-add-on group was far greater than that in the treated group (add-on vs. non-add-on, 1.07 ± 9.24 vs. -3.13 ± 7.90, *p* = 0.03). In terms of the absolute change in FVC between the groups, the treated group (29.00 ± 252.4 ml) lost significantly less FVC than that the non-add-on group did (-102.8 ± 235.2 ml) at the end of the 1-year follow-up period (*p* = 0.02). Furthermore, intragroup comparisons revealed that patients who were treated with antifibrotic drugs had similar FVC values [year 0 vs. year 1, 2750(1083) vs.2779(1093), *p* = 0.47] and pFVC values [year 0 vs. year 1, 83.30 ± 19.26 vs. 84.37 ± 20.26, *p* = 0.53] at the end of the follow-up period as at baseline. In contrast, those treated with conventional prednisone and immunosuppressants had a significantly lower FVC [year 0 vs. year 1, 2708 ± 748.6 vs.2605 ± 785.9, *p* < 0.01] and pFVC [year 0 vs. year 1, 85.61 ± 17.58 vs. 83.07 ± 19.15, *p* < 0.01] values after the 1-year follow-up. In addition, since a ≥ 5% change in %FVC is considered meaningful in clinical practice, we further analyzed the proportion of patients in each group whose pFVC change by 5% or more. There was no difference in the proportion of patients whose pFVC declined over 5% at the end of follow-up (*p* = 0.82), however, there were more patients with pFVC improvement in the add-on group (12(40.00%)) than those in the non-add-on group (9(16.07%)) (*p* = 0.02).

**Table 2:** Comparison of PFT between the two groups

Project	Add-on group *N* = 30	Non-add-on group *N* = 60*	Statistic value	*P* value
Primary outcome				
△pFVC(%)	1.07 ± 9.24	-3.13 ± 7.90	T = 2.20	0.03
Secondary outcome				
△pFEV1(%)	0.00(15.23)	-2.00(9.1)	U = 679	0.18
△FEV1(mL)	16.67 ± 234.7	-73.35 ± 188.9	T = 1.92	0.06
△FVC(mL)	29.00 ± 252.4	-102.8 ± 235.2	T = 2.41	0.02
△pTLC(%)	0.67 ± 10.03	-1.06 ± 7.07	T = 0.92	0.36
△TLC(mL)	109.7 ± 522.8	-98.70 ± 401.2	T = 2.04	0.04
△pDLCO(%)	-1.31 (13.44)	-9.66(12.15)	U = 437	< 0.01
△DLCO (ml/min`mmHg)	-0.01(0.99)	-0.40(0.79)	U = 544	0.01
△pFVC ≥ 5% decline	8(26.67%)	18(32.72%)		0.82
△pFVC ≥ 5% improvement	12(40.00%)	9(16.07.00%)		0.02
PPF at the end	20(66.7%)	31(51.7%)		0.16

*FEV1* forced expiratory volume in 1 s, *FVC* forced vital capacity, *DLCO* the single-breath diffusing capacity of the lung for CO adjusted by hemoglobin level a, *TLC* total lung capacity

*Four patients in the non-add-on group did not complete the 1-year pulmonary function test; therefore, the total number of patients included in PFT analysis for non-add-on group was 56

Further investigations of other PFT measurements throughout the follow-up period were also conducted (Table 2). The absolute change in DLCO (add-on vs. non-add-on, -0.01(0.99) vs. -0.40(0.79), *p* = 0.01) and pDLCO (add-on vs. non-add-on, -1.31 (13.44) vs. -9.66(12.15), *p* < 0.01) displayed huge difference between the groups. There was a significant difference in the absolute TLC change between the two groups (add-on vs. non-add-on, 109.7 ± 522.8 vs. -98.70 ± 401.2, *p* = 0.04). However, the change in the pTLC was not significantly different (*p* = 0.36). In addition, the changes in FEV1 (*p* = 0.06) and pFEV1 (*p* = 0.18) between the groups revealed no marked differences.

**Fig. 4:**
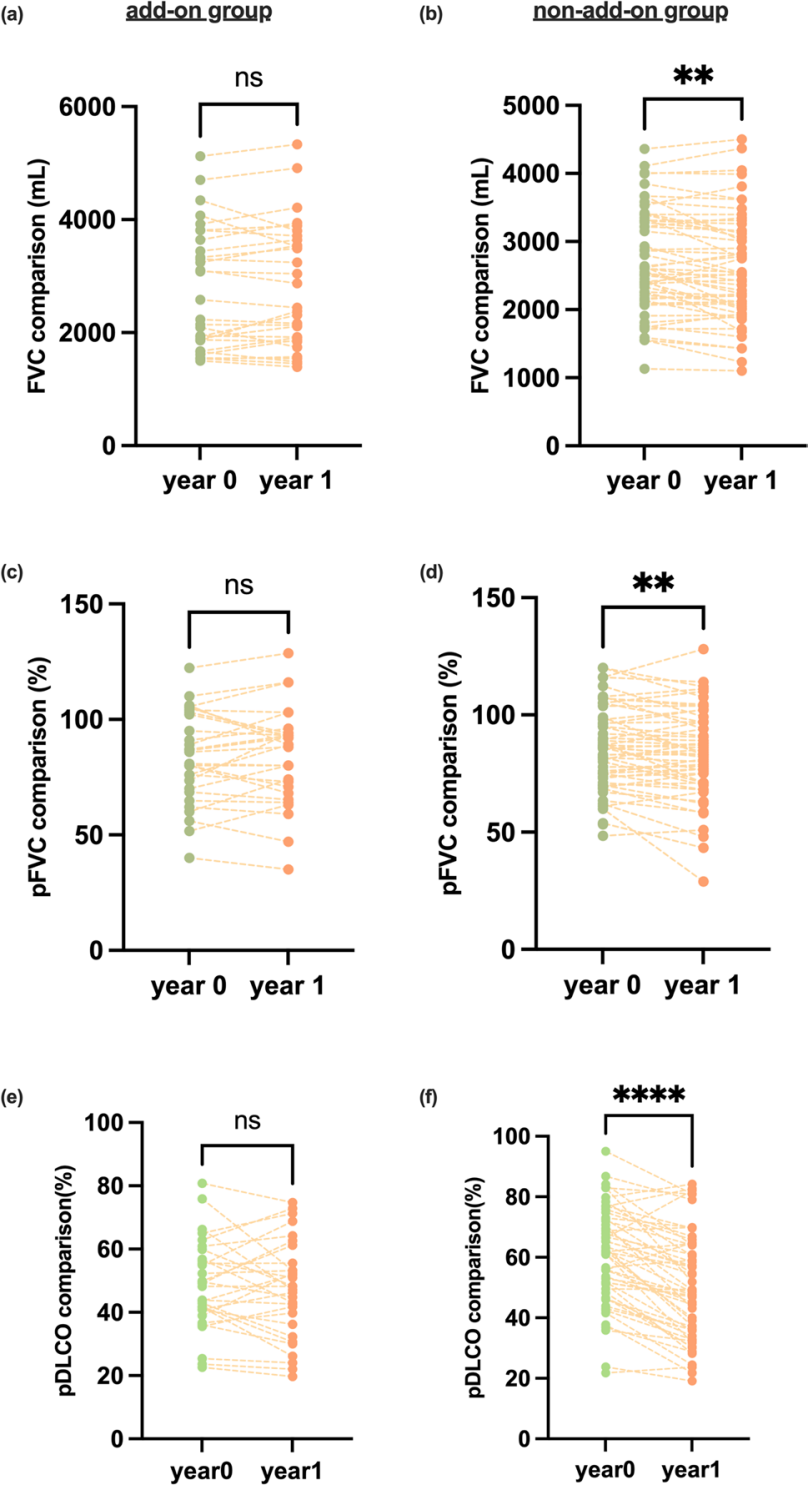
The comparison of FVC (**a**, **b**), pFVC (**c**, **d**), and TLC (**e**, **f**) in each group at the beginning and the end of the follow-up. *: *p* < 0.05, **: *p* < 0.01, ***: *p* < 0.001

Further intragroup comparisons revealed similar results (Fig. 4), with patients who received antifibrotic treatment preserving more DLCO and pDLCO (*p* = 0.90 and *p* = 0.43) than those in the non-add-on group did (*p* < 0.0001 and *p* < 0.0001), and the same phenomenon was observed in FVC (add-on vs. non-add-on, *p* = 0.09 vs. *p* < 0.01) and pFVC (add-on vs. non-add-on, *p* = 0.19 vs. *p* < 0.01). With respect to pTLC (add-on: *p* = 0.72; non-add-on: *p* = 0.28), and pFEV1 (add-on: *p* = 0.75; non-add-on: *p* = 0.04), intragroup comparisons of both groups revealed no significant differences. Furthermore, the proportion of patients who ultimately met the PPF criteria was similar between the two groups (*p* = 0.16).

### Medication treatment-associated outcomes

There was no significant difference between the two groups in terms of the infection rate (*p* = 1.00). Among the patients who were treated with antifibrotic drugs, 4 patients (13.33%) developed infections, 2 of whom (6.67%) had bacterial infections and 2 patients (6.67%) had COVID-19 infections. Eight patients (13.33%) experienced infections in the non-add-on group, including 4 patients (6.67%) had COVID-19 infection, 2 patients (3.33%) had bacterial infections, and 2 patients (3.33%) had oral candidiasis. Since these two antifibrotic drugs are associated with liver function impairment, we further focused on liver dysfunction during the follow-up period in the two groups. Only 2 patients (3.33%) in non-add-on group and none in the add-on group had elevated transaminase levels. With respect to the gastrointestinal tract, 3 patients in the add-on group and none in the non-add-on group complained of diarrhea. With respect to acute exacerbation risk, 3 patients (10.00%) and 4 patients (6.67%) experienced acute exacerbation of fILD during the 1-year follow-up in the add-on and non-add-on group, respectively (*p* = 0.68).

In addition, a sensitivity analysis using a time-dependent Cox model was conducted to address immortal time bias. The results are presented in Supplementary Table 2. Due to the limited number of treated patients (*n* = 13), this analysis was underpowered, and the hazard ratio for antifibrotic therapy was non-significant (HR = 1.01, 95% CI 0.47–2.19, *P* = 0.971). In addition, since the prevalence of immunosuppressive and glucocorticoids treatment in non-add-on group was far more than that in add-on group, we ran a univariate linear regression analysis to examine its potential confounding effect on pFVC (primary endpoint). The results demonstrated that neither glucocorticoid use (*p* = 0.81) nor immunosuppressant use (*p* = 0.94) showed a statistically significant association with the outcome.

## Discussion

To the best of our knowledge, this is the first comparative study to indicate that adding antifibrotic treatment to prednisone and immunosuppressive therapy in the treatment of ANCA-positive patients with fILD could ameliorate the annual decline in FVC and DLCO without additional adverse effects or acute exacerbations. As a result, we recommend considering antifibrotic drugs as a therapeutic option for patients with ANCA-fILD and propose high-quality prospective studies to further validate these findings.

According to findings of the University of California San Francisco cohort study, University of Chicago cohort study [17], and Japanese cohort study [7, 8], the prevalence of ANCA positivity in IPF patients approached 5–10%. However, only 26–33% of patients with ANCA-ILD present with systemic vasculitis, predominantly MPO-ANCA-positive patients, which is similar to our findings. That is, ANCA-ILD should not be simply considered the precursor of systemic vasculitis. Among ANCA-positive ILD patients [18], some may present pulmonary manifestations of AAV, whereas others may either progress to AAV with systemic involvement or remain ANCA-fILD without extrapulmonary disease. In this study, only 16.67% and 8.33% of patients in the add-on and non-add-on antifibrotic groups, respectively, eventually met the criteria for AAV by the end of the follow-up period, while most patients were diagnosed with isolated ANCA-fILD. Interstitial pneumonia with autoimmune features (IPAF) was first proposed and defined in 2015 [19], with ANCA positivity not included in its serological domain. Furthermore, there is a higher prevalence [9] of pulmonary fibrosis and a poorer response to corticosteroids or immunosuppressants among ANCA-ILD patients than among IPAF patients. These distinct clinical features position ANCA-ILD as a unique entity warranting focused research. According to current definitions, ANCA-ILD without systematic involvement (especially renal involvement) is classified as pulmonary limited vasculitis [18, 20]. Notably, several studies have revealed that the prognosis of patients with AAV-ILD is better than that of patients with ANCA-ILD, with the UIP pattern being a risk factor for poorer outcomes. As patients with ANCA-fILD have a poor response to immunosuppressants, Specks et al. [13, 21] recommends antifibrotic treatment ahead of immunosuppressant treatment. We reached a similar conclusion in our study: adding antifibrotic treatment for patients with ANCA-fILD could ameliorate the decrease in the annual FVC. As a result, we propose a combined therapeutic approach for ANCA-fILD involving the addition of antifibrotic drugs with corticosteroids and/or immunosuppressants. Furthermore, we attempted to address this bias quantitatively using a time-dependent Cox proportional hazards model. However, this analysis was profoundly underpowered due to the small number of patients who initiated antifibrotic therapy within the first year after diagnosis (*n* = 13) and the consequent low number of observed events. Therefore, our primary conclusions are rightly based on the analysis of continuous change in lung function parameters, which is a more robust and statistically efficient approach for the current sample size and study design. This analysis consistently demonstrated a signal of benefit associated with add-on therapy. This methodological challenge itself reflects a key characteristic of our cohort: the use of antifibrotic therapy in a real-world, early-intervention setting, often before the onset of rapid progression. In such a population with a slower baseline rate of decline, the time-to-event analyses may be less sensitive than direct measurement of functional change. The stabilization of lung function observed in our add-on group, using these direct measures, suggests a biologically plausible treatment effect in this specific clinical context.

While initially indicated for IPF, antifibrotic therapies have been successfully expanded to new applications following comprehensive clinical evaluation: progressive fibrosing interstitial lung disease and systemic sclerosis-ILD (SSc-ILD). The INBUILD trial [22] revealed that the application of nintedanib in the treatment of progressive fibrosing interstitial lung disease, also known as progressive pulmonary fibrosis, could reduce the likelihood of ILD progression irrespective of the underlying diagnosis. A total of 25.6% of the participants in the INBUILD trial presented with autoimmune factors. Furthermore, the SENSCIS trial [23] evaluated the efficacy of nintedanib in the treatment of SSc-ILD, revealing that the drug could slow the decrease in the adjusted annual rate of FVC. For pirfenidone, even though the primary endpoint was not evaluated due to inconsistency in home spirometry, the addition of pirfenidone in treating patients with progressive lung fibrosis, apart from IPF, tremendously decreased the decline in pFVC [24]. In summary, except for IPF, anti-fibrotic treatment is a promising option for interstitial lung disease patients with predominant pulmonary fibrosis. We reached a similar conclusion in our study: in fILD patients who were ANCA positive, adding antifibrotic treatment could ameliorate the progression of deterioration of pulmonary function. It is noteworthy that FVC and pFVC remained stable in the add-on group, suggesting that early antifibrotic intervention—even before meeting PF-ILD or PPF criteria—might suppress disease progression.

In regard to evaluating drug use, it is necessary to consider side effects. The common side effects of antifibrotic drugs include diarrhea, rash, abnormal liver function, and increased infection risk [25]. Several studies have noted the clinical use of antifibrotic drugs together with corticosteroids and/or immunosuppressants. However, these studies revealed no increased risk of adverse drug reactions or definitive contraindications to combination therapy. Similarly, according to our study, adding antifibrotics in the treatment of ANCA-fILD does not increase the risk of adverse drug reactions, infections, or acute exacerbations, indicating an acceptable safety profile for this therapeutic approach.

Our study has several limitations. First, as a single-center retrospective study, the detailed recording of the side effects observed during treatment might be insufficient. Second, owing to the limited sample size, we did not stratify different types of ANCAs further. Third, matching the baseline FVC%pred only in such a single-center study would inevitably introduce sampling bias. Fourth, a potential for immortal time bias exists due to the varying initiation times of antifibrotic therapy during the follow-up period. Therefore, additional multicenter, prospective, large-scale studies with subgroup analyses of ANCA types are needed to further validate our findings.

## Conclusion

In conclusion, we evaluated the effect of adding antifibrotics in the treatment of ANCA-fILD. The results suggest that the use of antifibrotics in the treatment of ANCA-fILD can preserve pulmonary function, with similar adverse risks as those associated with conventional treatment options.

## Supplementary Information

Supplementary Material 1.

## Data Availability

The dataset supporting the conclusions of this article is available through reasonable request to the first author Zhiyi Li (Email: [susie_zhiyi_li@outlook.com](mailto: susie_zhiyi_li@outlook.com) )
