# Supplementary material for: Efficacy of antifibrotic treatment for ANCA-positive fibrosing interstitial lung disease: a retrospective case‒control study
Source: BMC Pulm Med. 2026 Jan 22;26:78. doi: 10.1186/s12890-026-04109-1 (PMC12910929; doi:10.1186/s12890-026-04109-1)
Supplement: Supplementary file 1 — Supplementary Material 1. [file 12890_2026_4109_MOESM1_ESM.docx]

Supplementary table 1. Patients’ baseline characteristics before and after case-match

| Characteristic | Before matching | | | After matching | | |
| --- | --- | --- | --- | --- | --- | --- |
|  | Add-on group  (n=31) | Non-add-on group  (n=74) | P value | Add-on group  (n=30) | Non-add-on group  (n=60) | P value |
| Age | 66.00(10.00) | 65.00(11.25) | 0.90 | 66.00(11.25) | 64.50(11.75) | 0.86 |
| Gender | | | | | | |
| Male | 18(58.06%) | 40(54.05%) | 0.84 | 18(60.00%) | 30(50.00%) | 0.50 |
| Female | 13(41.94%) | 34(45.95%) |  | 12(40.00%) | 30(50.00%) |  |
| Type of ANCA antibody | | | | | | |
| p-ANCA | 22(70.97%) | 65(87.84%) | 0.04 | 22(73.33%) | 52(86.67%) | 0.15 |
| c-ANCA | 4(12.90%) | 7(9.46%) | 0.72 | 4(13.33%) | 5(8.33%) | 0.47 |
| MPO-ANCA | 9(29.03%) | 29(39.19%) | 0.38 | 8(26.67%) | 24(40.00%) | 0.25 |

Supplementary table 2. Time-dependent cox proportional hazards regression analysis for the risk of PPF

| Variable | Hazard ration | 95%CI | P value |
| --- | --- | --- | --- |
| Antifibrotic therapy | 1.01 | 0.47-2.19 | 0.97 |
